# Doublecortin-like kinase 1 expression associates with breast cancer with neuroendocrine differentiation

**DOI:** 10.18632/oncotarget.6386

**Published:** 2015-11-25

**Authors:** Yu-Hong Liu, Julia Y.S. Tsang, Yun-Bi Ni, Thazin Hlaing, Siu-Ki Chan, Kui-Fat Chan, Chun-Wai Ko, S. Shafaq Mujtaba, Gary M. Tse

**Affiliations:** ^1^ Department of Pathology, The Affiliated Baoan Hospital of Southern Medical University, Shenzhen, China; ^2^ Department of Anatomical and Cellular Pathology, The Chinese University of Hong Kong, Hong Kong; ^3^ Department of Anatomic Pathology, Centro Hospitalar Conde de Sao Januario, Macao, SAR, China; ^4^ Department of Pathology, Kwong Wah Hospital, Hong Kong; ^5^ Department of Pathology, Tuen Mun Hospital, Hong Kong; ^6^ Histopathology Section, Laboratory Department, King Abdullah Medical City, Makkah, Kingdom of Saudi Arabia

**Keywords:** doublecortin-like kinase 1, breast cancer, neuroendocrine differentiation, prognosis

## Abstract

Doublecortin-like kinase 1 (DCLK1), a microtubule associated kinase, has recently been proposed to be a putative marker for stemness and adverse prognosis in gastrointestinal cancers. However, it is not clear whether the protein also plays similar roles in breast cancer. Here, the expression of DCLK1 was analyzed in a large cohort of invasive breast cancers (IBC) by immunohistochemistry. DCKL1 was associated with favorable clinico-pathologic features, namely lower histologic grade, absence of lymphovascular invasion, fibrotic focus, necrosis and lower pN stage (*p*≤0.045). Additionally, independent significant correlations were found with estrogen receptor and neuroendocrine markers (*p* ≤0.019), implicating its relationship with IBC with neuroendocrine differentiation (IBC-NED). In the current cohort, IBC-NED showed worse outcome than luminal cancers without NED (hazard ratio=1.756, *p*=0.041). Interestingly, within the IBC-NED group, DCLK1 was found to be a good prognostic factor (hazard ratio =0.288, *p*=0.011). These findings were in contrast to those in gastrointestinal cancers, suggesting different functional roles of DCLK1 in different types of cancers. In clinical practice, NED is not routinely assessed; thus IBC-NED are not well studied. Its poor outcome and significant heterogeneity warrants more attention. DCLK1 expression could aid in the prognostication and management of this special cancer subtype.

## INTRODUCTION

Doublecortin-like kinase 1 (DCLK1) is a microtubule associated kinase, containing two doublecortin domains in the N-terminus for regulation of microtubule polymerization and a serine/threonine protein kinase domain in the C-terminus. It also shows substantial homology to Ca2+/calmodulin-dependent protein kinase. In between the N and C termini, there is a serine/proline-rich domain for mediating multiple protein-protein interactions [1]. Its function was first described in neuronal migration and development [2]. Recently, its expression has been found in colon cancers [3-5], pancreatic [6] and esophageal cancers [7].

DCLK1 has been proposed as a cancer stem cell marker for gastrointestinal cancers. DCLK1 specifically marked cancer stem cells (CSC) that self-renew and generate tumor progeny in *Apc^Min/+^* mice [8]. DCLK1 positive differentiated tuft cells can be activated by tissue injury and initiate colon cancer [9]. In pancreatic cancers, DCLK1 positive cells displayed increased sphere forming and tumor initiating capacity [10], and enhanced epithelial-mesenchymal transition (EMT), a process closely linked to the acquisition of stem cell properties, via regulation of microRNA biogenesis [4, 6, 11]. Interestingly, colorectal cancer with high DCLK1 expression had increased cancer specific mortality [5]. Upregulation of DCLK1 expression in blood circulation was found in chemoradiotherapy-treated colorectal cancer patients [12].

Aberrant DCLK1 expression was detected in IBC [13]. Knockdown of oncogenic miR-21 in IBC was accompanied by a decrease in DCLK1 expression [14]. Apart from these, no other information is currently available. It is not clear whether the aberrant DCLK1 expression contributed to IBC tumor aggressiveness as in gastrointestinal cancers. In addition, CSC heterogeneity exists in different breast cancer subtypes [15], and the commonly used CSC markers did not identify all CSC populations. It will be interesting to explore using DCLK1 as a CSC marker in breast cancer subtypes.

In this study, we evaluated the expression of DCLK1 in a large cohort of breast cancer by immunohistochemistry (IHC), its association with clinico-pathological features and other biomarkers (including CSC markers) expression, as well as the relationship with breast cancer outcome.

## RESULTS

**Figure 1:**
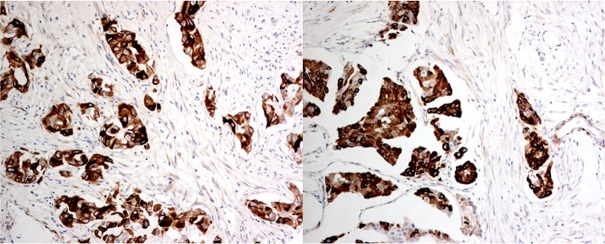
Representative immunohistochemical staining of DCLK1 (x400) High cytoplasmic immunoreactivity of DCLK1 in tumor cells but not in surrounding stroma. All micrographs were taken with a 40x objective, Nikon microscope equipped with a digital color camera and software.

A total of 1132 cases were included in this cohort. The mean patients' age was 54.6±12.7 (range 22-97) years. The mean tumor size was 2.67±1.52 (range 0.2-13.9) cm. One hundred and seventy three cases (15.3%) were grade I, 457 cases (40.4%) were grade II and 502 cases (44.3%) were grade III. Nine hundred and eighty seven cases were IBC of no special type (IBC-NST). There were 35 cases of invasive lobular cancers (ILC), 48 cases of breast cancers with medullary features, nine cases of mucinous cancers and eight cases of neuroendocrine cancers. The remaining 45 cases were of other miscellaneous histologic types, including micropapillary carcinoma, papillary carcinoma, tubular carcinomas, tubulo-lobular carcinoma and metaplastic carcinomas. Details of the clinico-pathologic features are summarized in Table 2. Overall, 418 cases (36.9%) were DCLK1 high and 513 cases (63.1%) were DCLK1 low. Representative staining is shown in Figure 1.

**Table 1:** Antibodies used for IHC analysis

Markers	Company	Clone	Dilution	Antigen retrival	Incubation condition (min, °C)	Assessment	cutoff
ER	Neomarkers	SP1	Pre-diluted	EDTA pH8	32,37	N	1%
PR	Ventana	IE2	Pre-diluted	EDTA pH8	32,37	N	1%
AR	Dako	AR441	1:100	EDTA pH8	56,37	N	1%
Ki67	Ventana	41912	Pre-diluted	EDTA pH8	32,37	N	14%
EGFR	Ventana	3C6	Pre-diluted	EDTA pH8	32,37	M	5%
HER2	Ventana	4B5	Pre-diluted	EDTA pH8	16,37	M	3+
CK5/6	Dako	D5/16 B4	1:40	EDTA pH8	32,37	C,M	5%
CK14	Neomarkers	LL002	1:100	EDTA pH8	32,37	C,M	5%
c-kit	Dako	104D2	1:300	EDTA pH8	32,37	C,M	5%
P63	Ventana	4A4	Pre-diluted	EDTA pH8	32,37	N	5%
Synaptophysin (SYN)	Novocastra	27G12	1:50	EDTA pH8	32,37	C,M	1%
Chromogranin (CG)	Biogene	MU-126-UC	1:200	EDTA pH8	32,37	C,M	1%
SOX2	Ventana	SP76	Pre-diluted	EDTA pH8	32,37	N	1%
vimentin	Dako	V9	1:2000	EDTA pH8	24, RT	C, M	10%
p-cadherin	BD transduction lab	56/p-cad	1:200	EDTA pH8	32,37	C,M	10%
CD44	Ventana	SP37	Pre-diluted	EDTA pH8	32, 37	M	5%
ALDH	BD transduction lab	44/ALDH	1:600	EDTA pH8	32, 37	C	5%
DCLK1	Abcam	Polyclonal	1:100	Citrate pH6	32, 37	C	IHC score 4

‘N’: nuclear; ‘C’: cytoplasmic; ‘M’: membraneous

**Table 2:** Correlation of DCLK1 expression with clinic-pathological features

		DCLK1				IHC score		
		lo	hi	Total	*p*-value	Mean (SD)	Median (IQR)	*P*-value
Grade	1	94	79	173	<0.001	3.3 (2.7)	3 (0-6)	<0.001
	2	259	198	457		3.0 (2.6)	3 (0-5)	
	3	361	141	502		2.3 (2.2)	2 (0-4)	
	Total	714	418	1132				
FF	Absence	519	323	842	0.045	2.8 (2.5)	2 (0-5)	0.208
	Presence	184	85	269		2.6 (2.4)	2 (0-4)	
	Total	703	408	1111				
necrosis	Absence	538	342	880	0.005	2.9 (2.5)	2 (0-5)	<0.001
	Presence	151	60	211		2.7 (2.5)	2 (0-4)	
	Total	689	402	1091				
EIC	Absence	565	325	890	0.680	2.8 (2.5)	2 (0-5)	0.741
	Presence	142	87	229		2.7 (2.5)	2 (0-4)	
	Total	707	412	1119				
LVI	Absence	472	309	781	0.004	2.8 (2.5)	3 (0-5)	0.034
	Presence	207	89	296		2.5 (2.2)	2 (0-4)	
	Total	679	398	1077				
pN	0	335	222	557	0.002	2.9 (2.5)	3 (0-5)	0.028
	1	220	120	340		2.6 (2.4)	2 (0-4)	
	2	84	39	123		2.5 (2.3)	2 (0-4)	
	3	60	19	79		2.1 (2.2)	2 (0-3)	
	Total	699	400	1099				
pT	1	280	182	462	0.078	2.9 (2.6)	3 (0-5)	0.442
	2	370	203	573		2.6 (2.4)	2 (0-4)	
	3	41	19	60		2.5 (2.3)	2 (0-4)	
	4	13	5	18		2.0 (2.0)	2 (0-3)	
	Total	704	409	1113				
Molecular	Lum A	283	253	536	<0.001	3.3 (2.6)	3 (0-6)	<0.001
	Lum B	210	110	320		2.7 (2.3)	2 (0-4)	
	HER2-OE	87	25	112		2.1 (1.9)	2 (0-3)	
	BLBC	52	16	68		2.0 (2.1)	2 (0-4)	
	5NP	75	10	85		1.2 (1.7)	0 (0-2)	
	Total	707	414	1121				
Age	Mean	54.1	55.5	54.6	0.266	-	-	-
	SD	12.2	13.5	12.7				
	Range	22-94	27-97					
Tumor size	Mean	2.71	2.60	2.67	0.076	-	-	-
	SD	1.48	1.57	1.51				
	Range	0.3-11.0	0.2-13.9					

“SD”: standard deviation; “IQR”: Interquartile range

### Correlation with clinico-pathologic features, biomarkers and molecular subtypes

High DCLK1 expression correlated with lower grade (*p* < 0.001), the absence of FF (*p* =0.045), the absence of necrosis (*p* = 0.005), the absence of LVI (*p* = 0.004) and lower pN stage (*p* = 0.002) but not age, EIC and pT stage (Table 2).

Among the 1121 invasive cancers with complete IHC data for molecular subtypes classification, 536 (47.8%) were luminal A, 320 (28.5%) were luminal B, 112 (10.0%) were HER2-OE and 153 (13.7%) were triple negative breast cancers (TNBC) (including 68 (6.1%) BLBC and 85 (7.6%) unclassified). The DCLK1 expression rate was 47.2% in luminal A, 34.4% in luminal B, 22.3% in HER2-OE and 17.0% in TNBC (23.5% in BLBC and 11.8% in unclassified). DCLK1 expression showed a differential expression in different molecular subtypes with the highest prevalence in luminal cancers (*p* < 0.001) (Table 2).

In line with that, DCLK1 also correlated positively with the expression of ER and PR (*p* < 0.001 for both). Additionally, DCLK1 correlated with expression of AR (*p* = 0.010), SYN (*p* < 0.001) and CG (<0.001) positively but negatively with HER2 (*p* = 0.001), Ki67 (*p* < 0.001), c-Kit (*p* = 0.034), CK5/6 (*p* = 0.030) and p-cadherin (*p* < 0.001). There was no significant correlation with EGFR, p63, CK14, CD44, ALDH1, vimentin and SOX2 (Table 3). By multivariate analysis, only LVI (OR=0.590, *p* = 0.001, 95% CI=0.427-0.817), ER (OR=2.316, *p* < 0.001, 95% CI=1.648-3.255), CG (OR=1.611, *p* = 0.019,95% CI=1.080-2.401) and SYN (OR=1.655, *p* < 0.001,95% CI=1.298-2.110) were found to be independent parameters associated with DCLK1 expression (Supplementary Table S1). Similar results were obtained when DCLK1 expression was analyzed as a continuous variable, except for FF (Tables 2-3 and Supplementary Table S2).

**Table 3:** Association of DCKL1 expression with biomarkers

		DCKL1				IHC score		
		lo	hi	Total	*p*-value	Mean (SD)	Median (IQR)	*P*-value
ER	Neg	263	65	328	<0.001	1.9 (2.0)	2 (0-3)	<0.001
	Pos	449	350	799		3.1 (2.5)	3 (0-5)	
	Total	712	415	1127				
PR	Neg	286	93	379	<0.001	2.1 (2.4)	2 (0-3)	<0.001
	Pos	420	322	742		3.1 (2.5)	3 (2-5)	
	Total	706	415	1121				
AR	Neg	444	235	679	0.043	2.5 (2.4)	2 (0-5)	0.004
	Pos	270	184	454		3.0 (2.4)	3 (0-5)	
	Total	714	419	1133				
EGFR	Neg	664	394	1058	0.297	2.8 (2.5)	2 (0-5)	0.793
	Pos	41	18	59		2.6 (2.2)	2 (0-5)	
	Total	705	412	1117				
HER2	Neg	552	358	910	0.001	2.9 (2.5)	3 (0-5)	0.002
	Pos	158	59	217		2.2 (2.0)	2 (0-4)	
	Total	710	417	1127				
Ki67	low	402	289	691	<0.001	3.0 (2.5)	3 (0-6)	<0.001
	high	302	126	428		2.4 (2.3)	2 (0-4)	
	Total	704	415	1119				
c-KIT	Neg	578	359	937	0.034	2.8 (2.5)	2 (0-5)	0.157
	Pos	124	53	177		2.5 (2.2)	2 (0-4)	
	Total	702	412	1114				
P63	Neg	674	399	1073	0.470	2.8 (2.5)	2 (0-5)	0.888
	Pos	30	14	44		2.6 (2.1)	2 (2-5)	
	Total	704	413	1117				
CK5/6	Neg	617	381	998	0.030	2.8 (2.5)	3 (0-5)	0.022
	Pos	85	33	118		2.2 (2.1)	2 (0-4)	
	Total	702	414	1116				
CK14	Neg	658	390	1048	0.465	2.8 (2.5)	2 (0-5)	0.313
	Pos	47	23	70		2.4 (2.1)	2 (0-4)	
	Total	705	413	1118				
SYN	Neg	659	329	988	<0.001	2.5 (2.4)	2 (0-4)	<0.001
	Pos	44	85	129		4.5 (2.4)	5 (4-7)	
	Total	703	414	1117				
CG	Neg	690	377	1067	<0.001	2.7 (2.4)	2 (0-5)	<0.001
	Pos	11	39	50		5.2 (2.3)	6 (4-7)	
	Total	701	416	1117				
SYN/ and CG	Neg	650	325	975	<0.001	2.5 (2.4)	2 (0-4)	<0.001
	Pos	47	88	135		4.5 (2.4)	5 (3-7)	
	Total	697	413	1110				
p-Cadherin	Neg	503	343	846	<0.001	3.0 (2.5)	3 (0-5)	<0.001
	Pos	192	64	256		2.1 (2.1)	2 (0-4)	
	Total	695	407	1102				
Vimentin	Neg	604	354	958	0.928	2.8 (2.5)	3 (0-5)	0.670
	Pos	94	56	150		2.7 (2.4)	2.5 (0-4)	
	Total	698	410	1108				
CD44	Neg	217	135	352	0.895	2.8 (2.5)	3 (0-5)	0.180
	Pos	105	67	172		3.1 (2.4)	3 (1-5)	
	Total	322	202	524				
ALDH	Neg	305	187	492	0.569	2.9 (2.5)	3 (0-5)	0.725
	Pos	20	15	35		3.1 (2.6)	2 (2-5)	
	Total	325	202	527				
SOX2	Neg	260	162	422	0.901	2.9 (2.5)	3 (0-5)	0.903
	Pos	64	41	105		2.9 (2.3)	3 (0-4.75)	
	Total	324	203	527				

“SD”: standard deviation; “IQR”: Interquartile range

### Relationship with clinico-pathologic features and biomarkers in IBC-NED

IBC-NED was defined by ≥ 1% expression of GC and/or SYN or showing morphological NED features [16-18]. All cases with morphologic NED features in fact showed NED marker expression. According to this criteria, 135 cases (12.1%) were classified as IBC-NED. Focal expression (1-49%) of either neuroendocrine markers was found in 81 cases and diffuse (≥50%) expression in 54 cases. Of these 135 cases, there were 112 IBC-NST. Others included 2 ILC, 4 carcinomas with medullary features, 3 mucinous carcinomas, 8 morphologic neuroendocrine carcinomas, 3 papillary carcinomas, 2 metaplastic carcinomas and one invasive micropapillary carcinoma. By molecular classification, 74 were luminal A, 54 were luminal B, 3 were HER2-OE and 3 were unclassified. IBC-NED (1% cutoff) were associated with the absence of necrosis (*p* = 0.028), the presence of LVI (*p* = 0.005), older age (*p* < 0.001) and luminal cancers (*p* < 0.001). Regarding biomarkers, it also correlated positively with ER and PR (*p* < 0.001 for both) and negatively with HER2 (*p* = 0.003), EGFR (*p* = 0.033), basal and EMT markers (including p63, c-kit, CK5/6, CK14, p-cadherin and vimentin; *p* ≤ 0.034) (Supplementary Table S3). Concentrating on IBC- NED with diffuse NED marker expression (50% cutoff), there were significant associations with necrosis, older age, ER, PR, HER2, c-kit, CK5/6 and p-cadherin, and these associations were similar to all IBC-NED (1% cutoff) (*p* ≤ 0.034). For LVI, EGFR and vimentin (Supplementary Table S3), the associations with all IBC-NED were not seen with IBC-NED (diffuse).

In IBC-NED, DCLK1 expression remained associated with lower grade (*p* < 0.001), lower pN stage (*p* ≤ 0.012), lower pT stage (*p* ≤ 0.042), PR positivity (*p* ≤ 0.019), HER2 negativity (*p* = 0.002), low Ki67 (*p* ≤ 0.004) and diffuse NED expression (*p* < 0.001) regardless categorical or continuous variables analyses (Table 4).

**Table 4:** Association of DCLK1 with clinico-pathological features and biomarkers in IBC-NED

		DCLK1				IHC score		
		lo	hi	Total	*p*-value	Mean	Median	*p*-value
Grade	1	2	14	16	<0.001	5.8 (1.8)	7 (4.5-7)	<0.001
	2	17	52	69		4.9 (2.2)	6 (3.5-7)	
	3	28	22	50		3.3 (2.4)	3 (1.5-6)	
	Total	47	88	135				
FF	Absence	34	75	109	0.079	4.6 (2.4)	5 (2.5-7)	0.366
	Presence	12	12	24		4.1 (2.4)	3.5 (2.5-6.5)	
	Total	46	87	133				
necrosis	Absence	39	76	115	0.440	4.5 (2.4)	5 (3-7)	0.463
	Presence	7	9	16		3.9 (2.9)	4 (0.5-7)	
	Total	46	85	131				
EIC	Absence	39	71	110	0.645	4.4 (2.5)	5	0.841
	Presence	7	16	23		4.7 (2.1)	5	
	Total	46	87	133				
LVI	Absence	20	59	79	0.006	4.8 (2.3)	5 (3-7)	0.093
	Presence	24	25	49		4.0 (2.5)	4 (2-7)	
	Total	44	84	128				
pN	0	16	47	63	0.001	4.9 (2.4)	6 (3-7)	0.012
	1	15	25	40		4.2 (2.3)	4 (2-6)	
	2	6	7	13		4.0 (2.5)	4 (2-6.5)	
	3	10	4	14		2.9 (2.1)	3 (1.5-4)	
	Total	47	83	130				
pT	1	14	39	53	0.028	5.0 (2.3)	6 (3-7)	0.042
	2	29	42	71		4.0 (2.5)	4 (2-6)	
	3	1	2	3		5.3 (2.1)	6 (4.5-6.5)	
	4	3	1	4		2.8 (2.5)	2.5 (1-4.5)	
	Total	47	84	131				
Molecular	Lum A	14	60	74	<0.001	5.2 (2.1)	6 (4-7)	<0.001
	Lum B	28	26	54		3.5 (2.4)	3 (2-6)	
	HER2-OE	3	0	3		1.7 (1.5)	2 (0-3)	
	BLBC	0	0	0		-	-	
	5NP	2	1	3		3.0 (3.0)	3 (0-3)	
	Total	47	87	134				
Age	Mean	56.6	59.7	58.7	0.249	-	-	-
	SD	13.0	14.0	13.7		-	-	
	Range	30-80	31-83					
Tumor size	Mean	2.86	2.58	2.68	0.208	-	-	-
	SD	1.35	1.27	1.31		-	-	
	Range	1.0-7.0	0.8-8.0					
Biomarkers								
ER	Neg	6	3	9	0.065	2.8 (2.5)	3 (0-5.5)	0.031
	Pos	41	85	126		4.6 (2.3)	5 (3-7)	
	Total	47	88	135				
PR	Neg	14	11	25	0.020	3.6 (2.2)	3 (2-5.5)	0.019
	Pos	33	77	110		4.7 (2.4)	5 (3-7)	
	Total	47	88	135				
AR	Neg	28	52	80	0.957	5.2 (2.1)	5 (3-7)	0.458
	Pos	19	36	55		4.7 (2.4)	5 (2-7)	
	Total	47	88	135				
EGFR	Neg	47	86	133	0.543	4.4 (2.4)	5 (3-7)	0.534
	Pos	0	2	2		5.5 (2.2)	5.5 (4-7)	
	Total	47	88	135				
HER2	Neg	37	84	121	0.002	4.6 (2.3)	6 (3-7)	0.002
	Pos	10	3	13		3.7 (2.4)	3 (0-4)	
	Total	47	87	134				
Ki67	low	21	63	84	0.002	4.9 (2.3)	6 (3.5-7)	0.004
	high	26	25	51		3.7 (2.4)	3 (2-6)	
	Total	47	88	135				
c-KIT	Neg	41	83	124	0.152	4.5 (2.5)	5 (2-7)	0.501
	Pos	6	5	11		4.2 (1.8)	3 (3-6)	
	Total	47	88	135				
P63	Neg	45	88	133	0.343	4.5 (2.4)	5 (3-7)	0.263
	Pos	1	0	1		2	2	
	Total	46	88	134				
CK5/6	Neg	46	86	132	0.546	4.4 (2.4)	5 (3-7)	0.238
	Pos	0	2	2		6.5 (0.7)	6.5 (6-7)	
	Total	48	88	134				
CK14	Neg	46	87	133	1.0	4.5 (2.4)	5 (3-7)	0.933
	Pos	1	1	2		4.5 (3.5)	4.5 (2-4.5)	
	Total	47	88	135				
CG/SYN	<50%	37	40	77	<0.001	3.7 (2.4)	4 (2-7)	<0.001
	≥50%	10	48	58		5.5 (2.1)	7 (5-7)	
	Total	47	88	135				
p-Cadherin	Neg	39	79	118	0.182	4.5 (2.4)	5 (3-7)	0.158
	Pos	8	8	16		3.8 (2.4)	3.5 (2-6)	
	Total	47	87	134				
Vimentin	Neg	46	81	127	0.421	4.4 (2.4)	5 (2-7)	0.359
	Pos	1	6	7		5.4 (1.5)	6 (4-7)	
	Total	47	87	134				
CD44	Neg	8	24	32	1.0	5.3 (2.2)	6 (3.5-7)	0.376
	Pos	5	13	21		4.7 (2.3)	5 (3.5-7)	
	Total	13	40	53				
ALDH1	Neg	12	38	50	1.0	5.0 (2.2)	6 4-7)	0.573
	Pos	1	2	3		5.7 (2.3)	7 (3-7)	
	Total	13	40	53				
SOX2	Neg	8	32	40	0.179	5.2 (2.2)	6 (4-7)	0.261
	Pos	5	8	13		4.5 (2.3)	4 (3-7)	
	Total	13	40	53				

“SD”: standard deviation; “IQR”: Interquartile range

### Relationship with outcome

Follow up data were available for 987 cases with a mean follow-up duration of 65.6 months (range 1–210 months). One hundred and forty nine cases (15.1%) had breast cancer mortality or relapse. Among them, 116 cases (11.8%) had breast cancer specific mortality. DCLK1 expression was associated with significantly better OS (log-rank=5.753, *p* = 0.016) and DFS (log-rank=12.104, *p* = 0.001). When segregating the cases into luminal and non-luminal cancers, DCLK1 expression was associated with better DFS significantly in luminal cancers (log-rank=5.883, *p* = 0.015) but not in non-luminal cancers (log-rank=0.389, *p* = 0.533).

**Figure 2:**
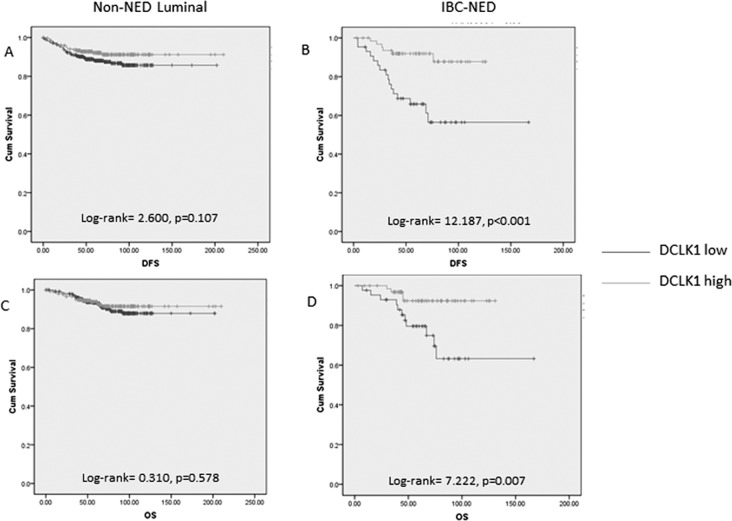
Kaplan-meier analysis of DFS and OS on non-NED luminal and IBC-NED cancers according to DCKL1 expression DFS in non-NED luminal cancers **A**. and IBC-NED **B**. with different level of DCLK1 expression was compared. OS in non-NED luminal cancers **C**. and IBC-NED **D**. with different level of DCLK1 expression was compared. DCKL1 expression was related to DFS and OS in IBC-NED but not non-NED luminal cancers.

**Figure 3:**
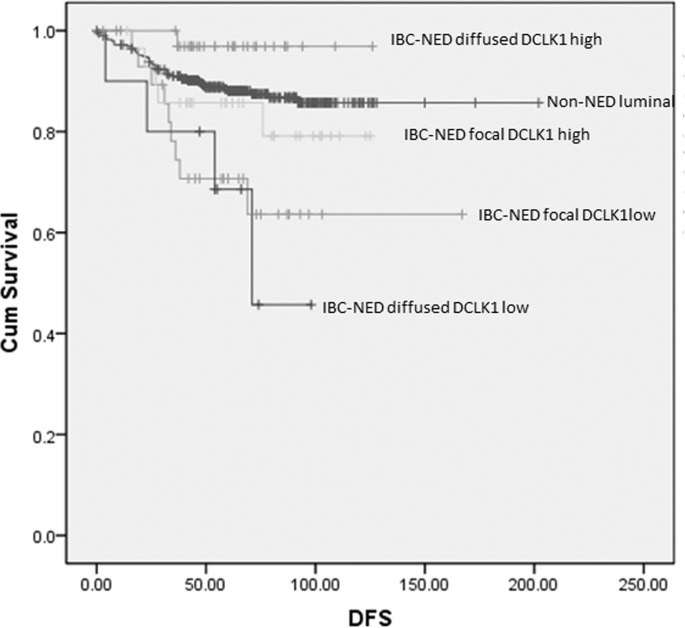
Kaplan-meier analysis on DFS of non-NED luminal and IBC-NED according to NED marker and DCKL1 expression DCKL1 low IBC-NED regardless of focal or diffused NED marker expression showed worse DFS compared to non-NED luminal cancers.

It appears that the association of DCLK1 with outcome in luminal cancers is mainly related to the relationship with IBC-NED, which are clustered within the luminal group of cancers. Analysis of the prognostic impact of DCLK1 in the IBC-NED cases and non-NED luminal cases found that DCLK1 expression was associated with better DFS (log-rank= 12.187, *p* < 0.001) and OS (log-rank=7.222, *p* = 0.007) in IBC-NED but not in non-NED luminal cancers (Figure 2). Recent investigations suggested adverse prognosis of IBC-NED [16, 19]. This study also showed worse OS (log-rank=4.658, *p* = 0.031) and DFS (log-rank=9.294, *p* = 0.002) in IBC-NED than luminal cancers without NED. The prognostic impact on DFS of IBC-NED was independent of ER, PR, HER2, Ki67, grade, age, tumor size and nodal involvement (HR=1.756, *p* = 0.041 with reference to non-NED luminal). Interestingly, those IBD-NED with worse outcome showed focal but not diffuse NED expression (supplementary figure 1S). As DCLK1 was associated with diffuse NED expression, we then compared the patients' parameters and outcome based on groupings stratified by different levels of neuroendocrine and DCLK1 expression. IBC-NED with low DCLK1 expression showed the lowest DFS rate, when compared to non-NED luminal cancers regardless of the neuroendocrine expression pattern (DCLK1 low/ neuroendocrine focal: log-rank=8.861, *p* = 0.003; DCLK1 low/ neuroendocrine diffuse: log-rank=7.211, *p* = 0.007) (Figure 3). Of note, DCLK1 was found also to have independent favorable prognostic impact on DFS of IBC-NED (HR=0.288, *p* = 0.011, 95%CI= 0.111-0.748) after adjustment of grade, age, tumor size, LVI, pN stage, HER2, Ki67, PR and neuroendocrine markers expression (Supplementary Table S4).

## DISCUSSION

In this study, the expression of DCLK1 in a large cohort of breast cancer was analyzed. In contrast to its cancer initiating roles in gastrointestinal tumors, DCLK1 expression in breast cancer did not appear to be related to stem cell features and aggressive behavior. No positive correlation was observed with other breast CSC markers. Nonetheless, DCLK1 was negatively correlated with grade, basal (c-kit and CK5/6) as well as EMT (p-cadherin) markers. DCLK1 was more frequently found in luminal cancers than basal-like and HER2-OE subtypes [15, 20, 21], associated with IBC-NED and potentially an independent favorable prognostic factor in these cancers.

The diagnosis of IBC-NED has been controversial. It was first defined by the presence of morphologic features similar to those of neuroendocrine tumors of the gastrointestinal tract or of the lung. However, classic neuroendocrine morphology is comparatively rare in breast cancer, thus its significance has been debatable for some time. Recently, a formal categorization of this tumor in WHO classification has been established. Apart from the morphologic features (including well-differentiated neuroendocrine tumors and poorly differentiated neuroendocrine carcinoma or small cell carcinoma), it has been defined with the expression of NED markers in over 50% of the tumor cell population in the 2003 WHO classification [22]. In the latest 2012 classification [18], it was revised to include all tumors expressing NED markers to a greater or lesser degree. The prognostic significance of NED per se in IBC-NED has also been uncertain, with reports suggested no effects on prognosis [23-25], better [26, 27] or worse [16, 19] prognosis. In the current study, the latest WHO criteria were adopted for diagnosis of IBC-NED [18]. In agreement with the others [16, 19, 28, 29], IBC-NED was mainly associated with luminal cancers. However, IBC-NED was not associated with low tumor grade or favorable outcome as would be expected for luminal cancers. This result corroborated with heterogeneity in IBC-NED, which encompass both low grade special subtypes and aggressive high grade cancers [17]. In contrast to the previous reports showing the poor prognosis of diffuse neuroendocrine markers expression [19], this study showed poorer outcome in patients with focal rather than diffuse NED expression in IBC-NED. One study applied similar criteria demonstrated similar poor outcomes in IBC with diffuse and focal neuroendocrine marker expression [16] compared to non-NED IBC cases. Of note, when the non-NED IBC were further classified into luminal and non-luminal cases, IBC-NED in fact showed an intermediate outcome, which was better than non-luminal but worse than non-NED luminal cancers. Possibly the heterogeneity in the cohort and variation in diagnostic criteria contributed to the variation in its prognostication.

As IBC-NED is heterogeneous, factors that further stratify IBC-NED into different prognostic groups could be useful for the management of these cancers. DCLK1 expression was found to be an independent favorable prognostic factor for DFS in IBC-NED regardless of the NED expression pattern. Currently, there are no specific recommendations for treatment of IBC-NED and these cases are treated as IBC-NOS. As IBC-NED are mostly luminal, they are treated with either endocrine therapy alone or together with adjuvant chemotherapy, with the latter depending on risk assessment. The additional independent prognostic impact of DCLK1 could be crucial for therapeutic decision to identify IBC-NED with favorable outcome, and sparing patients from chemotherapy. The mechanism of its good prognostic impact of DCLK1 in IBC-NED has not yet been explored. A recent report suggested that DCLK1 can antagonize Runx2 [30]. Runx2 has been shown to be a regulator of epithelial cell fate in mammary gland development and breast cancer [31]. Overexpression of Runx2 drove EMT–like changes in normal mammary epithelial cells, whereas its deletion in basal breast cancer cells inhibited cellular phenotypes associated with tumorigenesis [32]. In fact, in our preliminary study on Runx2 expression, we observed a significantly higher expression of basal marker in DCLK1loRunx2hi cases compared to the others (data not shown). Interestingly, there are also interactions between them in IBC-NED patients' outcome. Cases with DCLK1loRunx2hi expression were found to have significantly worse DFS than other subgroups (data not shown). Our data may suggest that DCLK1 could at least partly act via Runx2.

In summary, DCLK1 was found to be a good prognostic factor in breast cancer, particularly in IBC-NED. The result was in contrast to its tumor promoting roles in gastrointestinal cancers, suggesting different functional roles of DCLK1 in different type of cancers. In addition, using the current WHO classification, we found that IBC-NED showed a worse outcome, attributable in part to tumor heterogeneity. DCLK1 expression was shown to stratify IBC-NED into different prognostic groups. The findings could aid in the prognostication and management of this special type of IBC.

## MATERIALS AND METHODS

### Patients data

The histologic files of the three involved institutions were searched for IBC over periods of 2 (03-04), 4 (2002-05) and 7 (2003-09) years. All consecutive cases with excision specimens were included. All the specimens were formalin fixed, paraffin embedded and routinely processed. The 4 micron slides were stained with H&E and reviewed by two of the authors. The tumors were graded using modified Bloom and Richardson grading [16] and the histologic diagnosis was confirmed (WHO [17]). Invasive breast cancers with neuroendocrine differentiation (IBC-NED) were defined by the presence of neuroendocrine morphological features (neuroendocrine carcinoma) and the presence of neuroendocrine differentiation (NED) (neuroendocrine marker positivity) [17]. IBC was considered as having NED if ≥ 1% tumor cells showed any expression of GC and/or SYN or showing morphologic features of NED [18, 19]. In addition, lymphovascular invasion (LVI), the presence of extensive in situ components (EIC) and fibrotic focus (FF) were also evaluated as present or absent as previously reported [20]. Patient details and clinical information were retrieved from the medical records including patients' age, tumor size, pN stage, pT stage and patient outcome data. For the outcome data, overall survival (OS) was defined as the time interval from the date of initial diagnosis to the date of breast cancer related death. Disease free survival (DFS) was defined as the duration from the date of initial diagnosis to the first detection of breast cancer specific relapse or death. The study was approved by Joint Chinese University of Hong Kong- New Territories East Cluster clinical research ethics committee.

### Tissue microarray construction

Cellular areas of the tumors on H&E slides were chosen and the corresponding areas were taken from the paraffin blocks for tissue microarray (TMA) construction. Two 0.6 mm tissue cores were obtained from each case. One additional core was taken from available nodal metastases. The TMAs were assembled with a tissue arrayer (Beecher Instruments, Silver Springs, MD). Sixty two composite TMA blocks, each containing maximum 54 tissue cores, were constructed. Serial 4 micron sections were cut and transferred to Superfrost Plus glass slides (Menzel-Glaser, Germany). One section from each tissue array block was stained with H&E to confirm the presence of representative tumors in the TMA blocks.

### Immunohistochemistry and scoring

The TMA slides were assessed for the different groups of biomarkers, in addition to DCLK1. The first group were steroid hormone receptors (estrogen receptor (ER), progesterone receptor (PR), and androgen receptor (AR)). The second group were growth factor receptors (human epidermal growth factor receptor 2 (HER2), epidermal growth factor receptor (EGFR)) and a proliferation marker (Ki67). The other groups were neuroendocrine markers (chromogranin (CG) and synpatophysin (SYN)), basal markers (p63, c-kit, CK14 and CK5/6), cancer stem cell markers (CD44, SOX2 and ALDH1) and EMT markers (vimentin and p-cadherin). IHC of all markers was performed by BenchMark XT automated slide-staining instrument (Ventana, Arizona, USA) with Ultraview Universal DAB Detection Kit (Ventana, Arizona, USA) after deparaffinization, rehydration and antigen retrieval. After primary antibody incubation, the sections were incubated with anti-mouse horseradish peroxidase labeled polymer (Roche, Arizona, USA) for 30 min at room temperature, and then developed with diaminobenzidine. All slides were counterstained with hematoxylin. The TMA slides were scored for the intensity of staining in the nucleus, cytoplasm or membrane according to different antibodies by two of the authors blinded to the clinical information and the staining results of other markers. Details of the antibodies, antigen retrieval, staining conditions and scoring were listed in Table 1. For DCLK1 staining, the reactivity assessed was cytoplasmic. DCLK1 was assessed for both intensity and proportion of positively stained cells. The staining intensity was graded from 0 to 3 whereas the proportion of stained cells was scored on a scale of 0-4 (0= no detectable staining, 1= 1-25% positive cells, 2= 26-50% positive cells, 3=51-75% positive cells and 4= over 75% positive cells). An immunoscore was obtained by adding the intensity score and the percentage score. Positivity for DCLK1 was defined using the mean immunoscore as the cutoff. Immunoscore of 0-3 was regarded as negative and >3 as positive. Any discrepancies were resolved by reviewing at a multi-head microscope to reach a consensus.

The tumors were also classified into the 5 different molecular subtypes by immunohistochemical expression as surrogate as follows [21]:

Luminal A: ER+ and/or PR+, HER2-, CK5/6 +/− and Ki67 <14%
Luminal B: ER+ and/or PR+, CK5/6+/−, HER2+ or Ki67 ≥14%
HER2 over-expressed (HER2-OE): ER-, PR-, HER2+, CK5/6 +/−
Basal like breast cancers (BLBC): ER-, PR-, HER2-, (triple negative), CK5/6+ and/or EGFR+
Unclassified: ER-, PR-, HER2-, (triple negative), CK5/6- and EGFR-

### Statistical analysis

The findings were analyzed using the statistical software SPSS for Windows, Version 18. Chi-square analysis or Fisher's exact test were used to test for the association of DCLK1 expression with tumor grade, FF, LVI, EIC, pN, pT, molecular subtypes and biomarker expression. Mann-Whitney U test was used for analyzing the differences in patient's age and tumor size with DCLK1 expression. The relationship of DCLK1 as a continuous variable with various clinico-pathologic features and biomarker expression was also analyzed with Mann-Whitney U test (for categorical data) and spearman correlation (rs) (for continuous variables). Survival data were evaluated with Kaplan Meier analysis and Cox regression analysis using the backward Wald method. Statistical significance was established at *p* < 0.05.

## SUPPLEMENTARY MATERIAL TABLES AND FIGURE


