# Supplementary material for: Doublecortin-like kinase 1 expression associates with breast cancer with neuroendocrine differentiation
Source: Oncotarget. 2015 Nov 25;7(2):1464–76. doi: 10.18632/oncotarget.6386 (PMC4811473; doi:10.18632/oncotarget.6386)
Supplement: Supplementary file 1 [file oncotarget-07-1464-s001.pdf]

Doublecortin-like kinase 1 expression associates with breast cancer with neuroendocrine differentiation

Supplementary Material

Supplementary Table S1 Multivariate analysis on the association with DCKL1 expression

	OR	p-value	95% CI	
			Lower	Upper
LVI	0.590	0.001	0.427	0.817
Ki67	0.777	0.095	0.577	1.045
ER	2.316	<0.001	1.648	3.255
CG	1.611	0.019	1.080	2.401
SYN	1.655	<0.001	1.298	2.110

Only the last step of backward analysis was shown.
Factors included: age, grade, LVI, FF, necrosis, pN stage, Ki67, ER, PR, HER2, CK5/6, c-kit, AR, p-cadherin, CG and SYN

Supplementary Table S2 Correlation of DCLK1 expression with other biomarkers, age and tumor size (as continuous variables) in overall cohort and IBC-NED

		Overall	IBC-NED
Age	r_s	0.041	0.213
	p-value	0.167	**0.013**
	N	1133	135
Tumor size	r_s	-0.064	-0.119
	p-value	**0.033**	0.174
	N	1118	132
ER	r_s	0.225	0.306
	p-value	**<0.001**	**<0.001**
	N	1127	135
PR	r_s	0.236	0.423
	p-value	**<0.001**	**<0.001**
	N	1121	135
AR	r_s	0.074	0.061
	p-value	**0.014**	0.481
	N	1111	134
EGFR	r_s	-0.012	0.054
	p-value	0.700	0.536
	N	1117	135
HER2	r_s	-0.047	-0.208
	p-value	0.114	**0.016**
	N	1127	134
Ki67	r_s	-0.076	-0.215
	p-value	**0.011**	**0.012**
	N	1119	135
c-KIT	r_s	-0.046	-0.059
	p-value	0.129	0.495
	N	1114	135
P63	r_s	-0.026	-0.191
	p-value	0.388	**0.028**
	N	1116	133
CK5/6	r_s	-0.072	0.066
	p-value	**0.015**	0.448
	N	1116	134
CK14	r_s	-0.025	0.028
	p-value	0.396	0.747
	N	1118	135
SYN	r_s	0.252	0.412
	p-value	**<0.001**	**<0.001**
	N	1117	135
CG	r_s	0.202	0.266
	p-value	**<0.001**	**0.002**
	N	1117	135
p-Cadherin	r_s	-0.183	-0.177
	p-value	**<0.001**	**0.041**
	N	1102	134
Vimentin	r_s	-0.004	0.088
	p-value	0.886	0.315
	N	1108	134
CD44	r_s	0.043	-0.086
	p-value	0.321	0.542
	N	524	53
ALDH	r_s	0.013	0.081

	p-value	0.766	0.562
	N	527	53
SOX2	r_s	0.014	-0.132
	p-value	0.741	0.346
	N	527	53

Supplementary Table S3 Association with IBC-NED (cutoff of 1% and 50%) with clinico-pathological factors and biomarker

		NED (1% cutoff)			NED (50% cutoff)		
		Absence	Presence	p-value	Absence	Presence	p-value
Grade	1	147	16	0.401	155	11	0.003
	2	382	69		422	31	
	3	446	50		486	12	
FF	Absence	714	109	0.065	782	46	0.051
	Presence	243	24		261	7	
necrosis	Absence	749	115	0.028	822	48	0.013
	Presence	191	16		204	3	
EIC	Absence	766	110	0.382	834	45	0.328
	Presence	198	23		217	8	
LVI	Absence	683	79	0.005	731	37	0.848
	Presence	244	49		279	15	
pN	0	485	63	0.275	521	29	0.201
	1	294	40		322	16	
	2	107	13		117	4	
	3	64	14		76	2	
pT	1	400	53	0.934	432	25	0.345
	2	489	71		539	24	
	3	57	3		60	0	
	4	15	4		17	2	
Molecular	Lum A	451	74	<0.001	489	39	<0.001
	Lum B	264	54		306	15	
	HER2-OE	109	3		112	0	
	BLBC	68	0		68	0	
	5NP	81	3		84	0	
Age	Mean	54.1	58.7	<0.001	54.2	63.5	<0.001
	SD	12.5	13.7		12.6	13.2	
	Range	22-97	30-83		22-97	37-83	
Tumor size	Mean	2.68	2.68	0.491	2.68	2.48	0.558
	SD	1.55	1.31		1.54	1.0	
	Range	0.2-13.9	0.8-8.0		0.2-13.9	0.9-4.8	
Biomarkers							
ER	Neg	316	9	<0.001	325	1	<0.001
	Pos	659	126		738	53	
PR	Neg	351	25	<0.001	369	7	0.001
	Pos	619	110		688	47	
AR	Neg	584	80	0.898	637	32	0.929
	Pos	392	55		427	22	
EGFR	Neg	910	133	0.033	995	53	0.357
	Pos	57	2		58	1	
HER2	Neg	775	121	0.003	848	52	0.001
	Pos	201	13		214	2	
Ki67	low	587	84	0.693	633	40	0.037
	high	384	51		424	14	

c-KIT	Neg	800	124	0.008	879	51	0.034
	Pos	165	11		173	3	
P63	Neg	924	133	0.034	1009	54	0.268
	Pos	43	1		44	0	
CK5/6	Neg	853	132	<0.001	938	54	0.003
	Pos	116	2		118	0	
CK14	Neg	899	133	0.008	986	53	0.249
	Pos	68	2		69	1	
p-Cadherin	Neg	717	118	0.001	788	51	<0.001
	Pos	239	16		254	2	
Vimentin	Neg	818	127	0.002	899	51	0.101
	Pos	142	7		147	3	
CD44	Neg	312	32	0.306	333	15	0.197
	Pos	151	21		160	12	
ALDH1	Neg	435	50	0.782	464	25	0.692
	Pos	31	3		32	2	
SOX2	Neg	375	40	0.389	397	22	0.855
	Pos	91	13		99	5	
DCLK1	Low	650	47	<0.001	687	10	<0.001
	High	325	88		365	48	

Supplementary Table S4 Cox regression analysis on DFS in IBC-NED

	HR	95% CI		p-value
		Upper	Lower	
pN stage	2.050	1.408	2.984	<0.001
DCKL1	0.288	0.111	0.748	0.011

Factor included in initial step: grade, age, tumor size, LVI, pN, HER2 positivity, Ki67 positivity, ER positivity, PR positivity, focal NED, DCLK1 positivity

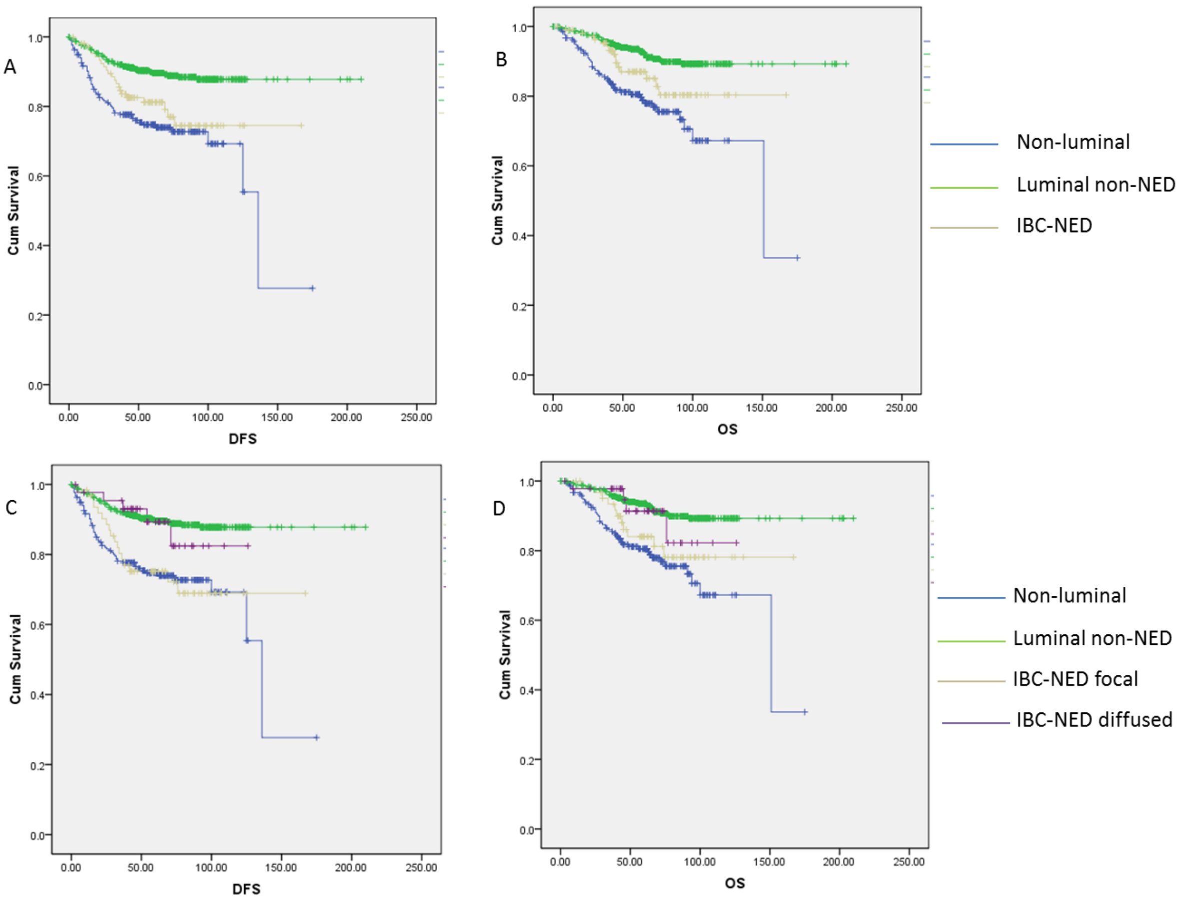

Figure S1: Kaplan-meier analysis on DFS and OS of IBC-NED compared to non-NED luminal and non-luminal cancers
